# Osteocytes contribute *via* nuclear receptor PPAR-alpha to maintenance of bone and systemic energy metabolism

**DOI:** 10.3389/fendo.2023.1145467

**Published:** 2023-04-18

**Authors:** Amit Chougule, Sudipta Baroi, Piotr J. Czernik, Emily Crowe, Mi Ra Chang, Patrick R. Griffin, Beata Lecka-Czernik

**Affiliations:** ^1^ Department of Orthopaedic Surgery, College of Medicine and Life Sciences, University of Toledo, Toledo, OH, United States; ^2^ Center for Diabetes and Endocrine Research, College of Medicine and Life Sciences, University of Toledo, Toledo, OH, United States; ^3^ Department of Molecular Medicine, UF Scripps Biomedical Research, University of Florida, Jupiter, FL, United States; ^4^ Department of Physiology and Pharmacology, College of Medicine and Life Sciences, University of Toledo, Toledo, OH, United States

**Keywords:** osteocyte, PPAR alpha, energy metabolism, bone mass, marrow adipocytes, osteoblasts, osteoclasts, fat beiging

## Abstract

**Introduction:**

The view that bone and energy metabolism are integrated by common regulatory mechanisms is broadly accepted and supported by multiple strands of evidence. This includes the well-characterized role of the PPARγ nuclear receptor, which is a common denominator in energy metabolism and bone metabolism. Little is known, however, about the role of PPARα nuclear receptor, a major regulator of lipid metabolism in other organs, in bone.

**Methods:**

A side-by-side comparative study of 5-15 mo old mice with global PPARα deficiency (α^KO^) and mice with osteocyte-specific PPARα deficiency (αOT^KO^) in order to parse out the various activities of PPARα in the skeleton that are of local and systemic significance. This study included transcriptome analysis of PPARα-deficient osteocytes, and analyses of bone mass and bone microarchitecture, systemic energy metabolism with indirect calorimetry, and differentiation potential of hematopoietic and mesenchymal bone cell progenitors. These analyses were paired with *in vitro* studies of either intact or silenced for PPARα MLO-A5 cells to determine PPARα role in osteocyte bioenergetics.

**Results:**

In osteocytes, PPARα controls large number of transcripts coding for signaling and secreted proteins which may regulate bone microenvironment and peripheral fat metabolism. In addition, PPARα in osteocytes controls their bioenergetics and mitochondrial response to stress, which constitutes up to 40% of total PPARα contribution to the global energy metabolism. Similarly to α^KO^ mice, the metabolic phenotype of αOT^KO^ mice (both males and females) is age-dependent. In younger mice, osteocyte metabolism contributes positively to global energetics, however, with aging the high-energy phenotype reverts to a low-energy phenotype and obesity develops, suggesting a longitudinal negative effect of impaired lipid metabolism and mitochondrial dysfunction in osteocytes deficient in PPARα. However, bone phenotype was not affected in αOT^KO^ mice except in the form of an increased volume of marrow adipose tissue in males. In contrast, global PPARα deficiency in α^KO^ mice led to enlarged bone diameter with a proportional increase in number of trabeculae and enlarged marrow cavities; it also altered differentiation of hematopoietic and mesenchymal marrow cells toward osteoclast, osteoblast and adipocyte lineages, respectively.

**Discussion:**

PPARα role in bone is multileveled and complex. In osteocytes, PPARα controls the bioenergetics of these cells, which significantly contributes to systemic energy metabolism and their endocrine/paracrine function in controlling marrow adiposity and peripheral fat metabolism.

## 1 Introduction

Peroxisome proliferator-activated receptors (PPARs) represent a group of nuclear receptors and transcription factors originally discovered to control biogenesis of peroxisomes. PPARs are also responsible for regulation of glucose and fatty acid metabolism, with PPARγ being a prominent target for TZDs, antidiabetic drugs, and insulin sensitizers, whereas PPARα is a pharmacologic target for fibrates, a class of drugs used to treat dyslipidemia. The third member of this family, PPARβ/δ is emerging as an essential factor in regulation of energy metabolism in muscles, and therapies to target this nuclear receptor to combat sarcopenia are in development. All three PPARs are expressed in bone cells and, by a virtue of the fact that bone and energy metabolism are inherently linked, they may play an important role in regulation of bone homeostasis.

While the role of PPARγ in regulation of bone homeostasis has been extensively studied in animals and humans, the role of PPARα in bone has not been systematically studied and available findings are not conclusive. Studies by Wu et al. did report observations of enlarged marrow cavities in PPARα-null mice; however, differentiation of bone marrow stroma cells toward osteoblasts or adipocytes was not found to be affected (1). Clinical evidence on the effect of fibrates on bone is limited. A small number of animal studies suggest that there is positive effect of fibrates on the skeleton and protection from TZD-induced bone loss (2–4).

The PPARα nuclear receptor monitors intracellular non-esterified fatty acid (NEFA) levels, which upon binding activate its transcriptional activities regulating expression of a number of genes involved in lipid metabolism and storage. PPARα is a major regulator of energy production in the process of fatty acid β-oxidation. It is highly expressed in metabolically active tissues such as those of the liver, heart, and muscle, which rely on fatty acid catabolism to fulfill energy requirements. In the liver, PPARα promotes fatty acid uptake and ketogenesis by regulating expression of genes involved in clearance of very low-density lipoprotein, fatty acid activation and transport into the mitochondria, and peroxisomal and mitochondrial β-oxidation, as well as several genes coding for enzymes in the mitochondrial respiration chain (5, 6). The role of PPARα in ketogenesis, a critical adaptive process during fasting, involves an FGF21-inducible effect on the expression of lipases required for generation of ketone bodies in adipose tissue; these serve as an energy source for other tissues (7, 8). Mice lacking PPARα develop liver steatosis during fasting (9) and obesity with aging, and are protected from high fat diet-induced insulin resistance (10). It is believed that the “Randle cycle,” a biochemical process involving competition for uptake and oxidation between glucose and fatty acids, plays a role in this effect (11).

It is well documented that bone metabolism relies on glucose, which provides energy to osteoblasts in the process of aerobic glycolysis (12). However, although the importance of lipids for bone growth and health has been acknowledged clinically, significant advances have only recently been made in understanding lipid metabolism in bone [reviewed in (13)]. First, uptake of fatty acids and postprandial lipoprotein uptake in bone are substantial and comparable or even greater to that occurring in muscle and in the heart (14, 15). Second, fatty acid uptake is essential for osteoblast function (16, 17). Third, fatty acid oxidation is required for postnatal bone acquisition and bone repair (18, 19). Most recently, it has been shown that fatty acid oxidation is also essential for osteoclast development (20). However, fatty acid metabolism in osteocytes, the most abundant cells in bone (21), has not been systematically addressed as yet.

Here, we present a study on the role of the PPARα nuclear receptor in bone and the first piece of evidence for an essential contribution of osteocytes, under PPARα control, to systemic energy metabolism and the development of bone marrow adipose tissue.

## 2 Materials and methods

### 2.1 Animals

To generate the αOT^KO^ mouse model, PPARα was deleted specifically in osteocytes using the Cre-Lox system. 10kb DMP1^cre^ mice (B6N.FVB-Tg (Dmp1-cre)1Jqfe/BwdJ), in which Cre expression is driven by the DMP1 promoter in osteocytes, were obtained from the Jackson Laboratory (Bar Harbor, ME). PPARα^fl/fl^ mice, which have loxP nucleotide sequences flanking exon 4 of PPARα, were obtained from the University of Toulouse, France (22). Mice from F3 progeny of DMP1^cre^ and PPARα^fl/fl^ crosses incorporated the desired osteocyte-specific PPARα knock-out, and are referred to as αOT^KO^. In all experiments, the control group (Ctrl) consisted of mice with either the PPARα^fl/fl^ or the DMP1-Cre genotype, after it was established that these two parental strains did not differ in analyzed parameters relating to bone and metabolic phenotype. Global PPARα KO (PPARα ^−/−^ 129S4/SvJae-PPARα ^tm1Gonz^/J) and adequate WT mice were purchased from the Jackson Laboratory. In PPARα KO (α^KO^) animals, Neo^R^ cassette replaced 83 bp of coding sequence in exon 8.

The colonies of α^KO^ and αOT^KO^ mice were maintained at the University of Toledo Health Science Campus. All animals were housed under a 12-h dark–light cycle and had free access to standard chow (Harlan Teklad 2016, Haslett, MI). Animal treatment and care protocols conforming to the National Institutes of Health Guidelines were followed under the University of Toledo Institutional Animal Care and Utilization Committee protocol. All presented experiments were performed on 5- to 15-month-old skeletally mature male and female mice.

### 2.2 Bone analysis

Microcomputed tomography (micro-CT) of the tibiae was performed using the μCT-35 system (Scanco Medical AG, Bassersdorf, Switzerland), as previously described (23). Briefly, scans were performed at 70 kVp energy and 113 μA intensity, and using 7-μm nominal resolution. Images of trabecular bone were segmented at a 260-threshold value using the per-mille scale with the three-dimensional noise filter set to sigma = 0.8 and support = 1.0. The analysis of bone microstructure conformed to recommended guidelines (24).

For lipid evaluation, decalcified bone specimens were stained for 1 h in solution containing 2% osmium tetroxide prepared in 0.1 M sodium cacodylate buffer, pH 7.4, according to the protocol (25). Staining was carried out in an exhaust hood and away from light due to osmium tetroxide toxicity and light sensitivity. Images of lipid depositions were acquired at 70-kVP and 113-μA and using 12-μm nominal resolution. Image segmentation was carried out under global threshold conditions by applying a grayscale threshold of 460–1000 using the per-mille scale, with the three-dimensional noise filter set to sigma = 1.2 and support = 2.0. Lipid volumes were calculated directly from individual voxel volumes in three-dimensional reconstructions.

### 2.3 Bone histomorphometry

To enable dynamic bone histomorphometry, mice were intraperitoneally injected with 20 mg/kg calcein (Sigma) 8 and 2 days before sacrifice, and undecalcified tibiae were fixed in 70% ethanol, embedded in methyl methacrylate, and sectioned. Images were recorded using a Zeiss Axiovert 40 CFL fluorescence microscope (Carl Zeiss Microscopy, Thornwood, NY) equipped with a Micropublisher 3.3 Megapixel Cooled CCD color digital camera (QImaging, Surrey, BC, Canada). Analysis of BFR was confined to the secondary spongiosa of the proximal tibia and was performed using the Nikon NIS-Elements BR3.1 system. The measurements were collected with a 40× objective lens (numerical aperture: 0.5) from 6 representative fields per bone sample. The terminology and units employed were those recommended by the Histomorphometry Nomenclature Committee of the American Society for Bone and Mineral Research (26). For static histomorphometry, bone sections were deplastinized; this was followed by von Kossa or TRAP staining for counting of osteoblasts or osteoclasts, respectively.

### 2.4 Genotyping

Genomic DNA samples were isolated from bone, liver, muscle, iWAT, and gWAT using Tri Reagent (Molecular Research Center, OH) and according to the protocol provided. 0.25 µg of genomic DNA was used for PCR amplification with a thermocycling protocol consisting of 3 min at 94°C, 35 cycles of 30 s at 94°C, 1 min at 50°C, and 1 min at 72°C. As in a previous study performed by Montagner et al. (22) to detect deletion of PPARα from genomic DNA, primers designed on both sides of PPARα exon 4 were used (**Supplementary Table 1**). Amplified DNA products were run on 1% agarose gel with 0.002% ethidium bromide.

### 2.5 Western blot analysis

Proteins isolated from femoral cortical bone using Tri Reagent were resolved by SDS-polyacrylamide gel electrophoresis and transferred to Immobilon-FL membranes. Membranes were blocked at room temperature for 1 h in TBS (10 mm Tris-HCl, pH 7.4, 150 mm NaCl) containing 3% BSA, then incubated with primary antibody overnight at 4°C. After 3 washes in TBST (TBS plus 0.1% Tween 20), membranes were incubated with infrared anti-rabbit (IRDye 800; green), anti-goat (IRDye 800; green), or anti-mouse (IRDye 680; red) secondary antibodies (LI-COR Biosciences, Lincoln, NE) at 1:10,000 dilution in TBS for 2 h at 4°C. Immunoreactivity was visualized and quantified *via* infrared scanning using the Odyssey system (LI-COR Biosciences). Antibodies against PPARα (sc-1982) and Hsp90 (sc-8262) were obtained from Santa Cruz Biotechnology (Santa Cruz, CA).

### 2.6 Cell culture

Bone marrow stromal cell (BMSC) cultures were established from femur marrow aspirates and differentiated as described (27). In brief, bone marrow cells were grown in the presence of beta-glycerol phosphate (10 mM) and ascorbic acid (0.2 mM) for osteoblastic differentiation for 26 days, and in the presence of rosiglitazone (1µM) for adipocytic differentiation for 3 days, after an initial 10 days of growth in basal media. The total number of colonies (CFU-F) and the number of adipocytic colonies (CFU-AD) or osteoblastic colonies (CFU-OB) were quantified after 13 or 26 days of culture, respectively. Floating, non-adherent cells were harvested after 24 h and seeded at a density of 2×10^5^/cm^2^ onto a 48-well plate with the medium supplemented with M-CSF (50 ng/ml) and RANKL (50 ng/ml) (R&D System, Minneapolis, MN). After 6 days of growth, cultures were stained for TRAP5b using the Leukocyte Acid Phosphatase (TRAP+) kit (Sigma) (27, 28).

### 2.7 Extraction of osteoblast- and osteocyte-enriched fractions from femoral bone

Cell fractions enriched in osteoblasts or osteocytes were isolated *via* sequential collagenase digestion of femoral bone according to a previously described protocol (29). In brief, isolated femurs were cleaned and scraped to remove soft tissues and periosteum, respectively. Bone marrow was spun down and epiphyses were removed. Bone diaphyses were sequentially digested in buffers supplemented with collagenase or EDTA. Fraction number 3 (which is an osteoblastic fraction) and fraction number 6 (an osteocytic fraction) were lysed with Tri Reagent and stored at -80°C. Total RNA was extracted from the samples according to the manufacturer’s recommended protocol.

### 2.8 Gene expression analysis using quantitative real-time PCR

Half microgram of the total RNA (by μg) was converted to cDNA using the Verso cDNA synthesis kit (Thermo Fisher Scientific, Waltham, MA). PCR amplification of the cDNA was performed *via* quantitative real-time PCR using the TrueAmp SYBR Green qPCR SuperMix (Smart Bioscience, Maumee, OH) and processed with the StepOne Plus System (Applied Biosystems, Carlsbad, CA). The thermocycling protocol consisted of 10 min at 95°C, 40 cycles of 15 s at 95°C, 30 s at 60°C, and 20 s at 72°C, followed by a melting curve step at temperatures ranging from 60 to 95°C to ensure product specificity. Relative gene expression was measured *via* the comparative CT method, using 18S RNA levels for normalization. Primers were designed using Primer-BLAST (NCBI, Bethesda, MD) and are listed in **Supplementary Table 1**.

### 2.9 Next-generation sequencing

Osteocytic fractions isolated from femora of 6-month-old WT or α^KO^ animals according to the protocol described in section 2.7 were plated on collagen-coated 100 mm plates supplemented with 5% FBS, 5% CS, and 1% PS media. After 6 days of growth, old media were replaced with fresh media. On 10^th^ day, osteocytes were collected *via* scraping, while the media enriched with osteocyte secretome (conditioned media) were used to grow WT BMSC for analysis of their pro-adipocytic effect in recipient cells. From donor osteocytes, RNA was extracted using the Qiagen RNeasy mini kit as per the manufacturer’s recommended protocol. The samples were processed and sequenced using a NextSeq 500 instrument at Scripps Research Institute, Jupiter, Florida. The overall quality of the reads was high, with no decrease over the course of sequencing. Principle Component Analysis (PCA) of the top 500 genes differentially expressed in the experimental groups showed biological variation between groups. Differentially expressed transcripts (p<0.05) were identified through pairwise comparison of samples from both groups using R version 3.4.0 and deseq2 version 1.16.1. To reduce false positives, the more conservative threshold of adjusted p-value < 0.05 was applied (p_adj_). Analysis of the raw data acquired from NGS in terms of differentially expressed transcripts considered base mean (transcript number), fold change (relative expression), and raw and adjusted p-values (significance). Raw data files are deposited in the NCBI Gene Expression Omnibus (GEO) repository, submission number: GSE225595. DAVID (The Database for Annotation, Visualization and Integrated Discovery) Bioinformatic Resources 6.8 was used for functional annotation and gene functional classification of up- or downregulated genes.

### 2.10 siRNA silencing of PPARα in MLO-A5 cells (αA5^KD^ cells)

PPARα silencing was achieved using *Pparα* siRNA (Santa Cruz Biotechnology), with DsiRNA NC1 (Integrated DNA Technologies, Coralville, IA) as a negative control. RNA oligonucleotides were delivered to MLO-A5 cells using the X-treme Gene siRNA Transfection Reagent (Roche; Cat#04476093001) according to a protocol provided by the manufacturer. αA5^KD^ cells and their scrambled (Scrl) control were used for assays 36 h after transfection.

### 2.11 Seahorse Mito Stress test

For Mito Stress testing, αA5^KD^ cells and their scrambled controls were plated 6 h before the assay onto Seahorse cell culture microplates (Agilent Technologies, Santa Clara, CA; Cat#101085-004). The four corner wells of the plate were used as negative controls. The assay medium consisted of Seahorse XF base medium without phenol red (Agilent Technologies, Cat#103335-100), supplemented with glucose (25 mM), sodium pyruvate (1 mM), and glutamine (2 mM). Oligomycin (1 µM), FCCP (2 µM), and rotenone/antimycin (0.9 µM) were used as stressors (Agilent Technologies, Cat#103015-100). Oxygen consumption rate (OCR) and extracellular acidification rate (ECAR) were measured at basal level and after addition of different stressors using a Seahorse XFe96 analyzer (Agilent Technologies). Assay operation software Wave version 2.6.1 was used to run the assay and collect the data. For normalization, live cells stained with Hoechst 33342 (1:2,000 dilution) (ThermoScientific, Cat#62249) were counted using a Cytation 5 plate reader (BioTek Instruments - Agilent). Values obtained from the Mito Stress test were normalized per 1,000 cells.

### 2.12 Indirect calorimetry

Mice were evaluated in calorimetric cages (CLAMS; Columbus Instruments, OH) for 4 days with free access to food and water. The mice were housed individually under controlled room temperature and humidity, and exposed to an alternating cycle of 12 h of light followed by 12 h of dark. After adaptation for 1 day, measures of O2 consumption (VO2), CO2 production (VCO2), respiratory exchange ratio (RER), heat production, and physical activity in the horizontal and vertical planes were collected for 3 consecutive days to determine energy expenditure. These data were obtained for each mouse, grouped according to genotype, and analyzed on an hourly basis or according to light vs. dark portion of the cycle. Respiratory exchange ratios (RERs) were calculated as the ratio of CO2 production to VO2 consumption. Total food and water consumption was measured during the experimental period.

### 2.13 Statistical analysis

Data are presented in the form mean ± SD and were analyzed using the statistical analysis software GraphPad Prism 9. Parametric, unpaired, two-tailed Student’s t-tests were conducted to compare means between two groups. A p-value of 0.05 or lower was considered to represent statistical significance.

## 3 Results

### 3.1 PPARα is expressed in osteocytes and regulates multiple signaling pathways

**Figure 1:**
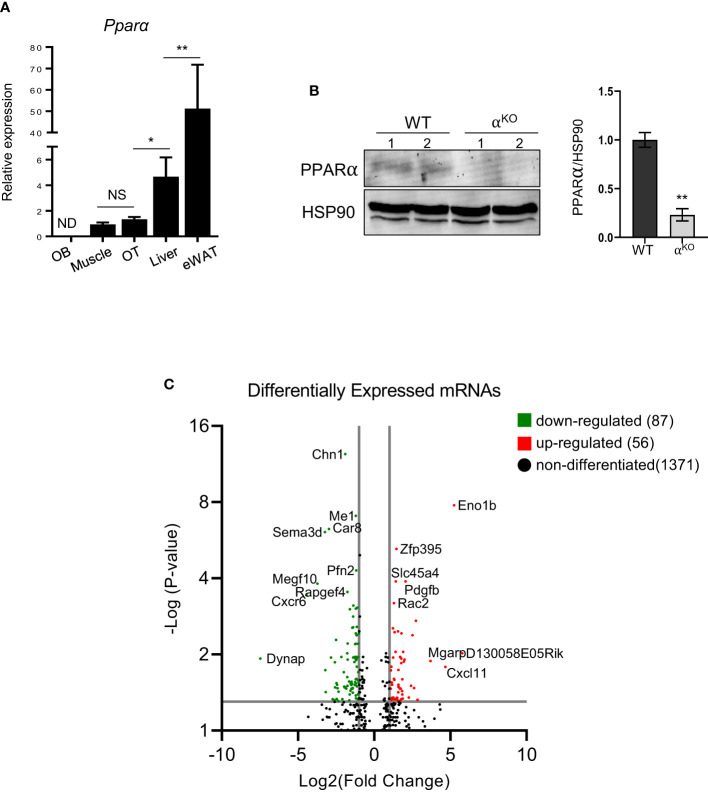
PPARα is expressed in osteocytes and controls their transcriptomics. **(A)** *Pparα* mRNA expression in endosteal osteoblasts (OB), soleus muscle, cortical osteocytes (OT), liver, and epidydimal white adipose tissue (eWAT) (n=4 mice per group). **(B)** Western blot analysis of PPARα protein level in cortical bone of WT and α^KO^ mice. Fifty µg of protein extracts were loaded per lane and PPARα band density was normalized to the HSP90 protein band. Normalized densities are shown on the accompanying graph (n=2 mice per group). Statistical significance was tested using a parametric unpaired Student’s t test. *p < 0.05, **p < 0.01. **(C)** Volcano plot of differentially expressed transcripts in osteocytes isolated from femoral cortical bone of α^KO^ mice as compared to WT osteocytes. The graph shows upregulated (red dots) and downregulated (green dots) transcripts with adjusted p values (p_adj_) < 0.05.

Compared to tissues whose metabolism relies on PPARα, *Pparα* mRNA expression in osteocytes isolated from murine femora was at a level similar to that observed in soleus muscle but much lower than that observed to liver tissue and eWAT (**Figure 1A**). Notably, *Pparα* expression was undetected in endosteal osteoblasts isolated from the same femoral bone (**Figure 1A**). Western blot analysis confirmed the presence of PPARα protein in control osteocytes and its absence in osteocytes isolated from femoral bones of α^KO^ mice (**Figure 1B**).

Analysis of the osteocyte transcriptome showed that PPARα controls a large number of transcripts coding for signaling proteins. Of 681 differentially expressed transcripts with p-value <0.01, 377 were downregulated and 304 were upregulated in PPARα-deficient osteocytes. Functional clustering identified several signaling pathways related to osteogenesis and glucose/fatty acid metabolism as being dysregulated in the absence of PPARα (**Table 1**, **Supplementary Table 2**). This included a number of downregulated genes in the Hippo, TGFβ/BMP, WNT, and Regulation of Stem Cell signaling pathways, while upregulated functional clusters included glucose, insulin, and fatty acid metabolism. The upregulated apoptosis cluster consisted of several transcripts coding for members of the BCL family and related inhibitors of apoptosis, suggesting protection from apoptosis in osteocytes deficient in PPARα.

**Table 1:** KEGG pathway analysis of transcriptomes of PPARα-deficient primary osteocytes.

Pathway	No. of Genes Affected	Downregulated (↓) Upregulated (↑)
Hippo signaling	22	↓
Signaling pathways regulating pluripotent stem cells	19	↓
Rap1 signaling	19	↓
TGF-beta signaling	10	↓
PI3K-Akt signaling	24	↓
Wnt signaling	12	↓
Focal adhesion	15	↓
MAP kinase signaling	19	↑
Insulin resistance	10	↑
Glycolysis/Gluconeogenesis	7	↑
Jak-STAT signaling	11	↑
TNF signaling	9	↑
FoxO signaling	10	↑
Apoptosis	6	↑
Insulin signaling	10	↑

More stringent analysis using adjusted p-values (p_adj_ < 0.05) indicated 56 upregulated and 87 downregulated transcripts in PPARα-deficient osteocytes (**Figure 1C**). Among the most significantly upregulated and potentially most relevant were transcripts for glycolytic enzyme enolase 1β (*Eno1b*) (upregulated 28-fold; p_adj_=1.68×10^-8^); sucrose transport solute carrier (*Slc45a4*) (upregulated 1.9-fold; p_adj_=0.0001); Rac family small GTPase 2 (*Rac2*), which may augment production of reactive oxygen species (upregulated 1.7-fold; p_adj_=0.0006); and mitochondria-localized glutamic acid-rich protein (*Mgarp*), which is involved in maintenance of mitochondrial abundance and morphology (upregulated 13.7-fold; p_adj_=0.013). Among the most significantly downregulated and potentially most relevant were transcripts for protein promoting activation of AKT1 (*Dynap*) (downregulated 56-fold; p_adj_=0.012); cell adhesion protein (*Megf1*) (downregulated 13.8-fold; p_adj_=0.0002); and several transcripts coding for proteins involved in neuron development, such as chimerin 1 (*Chn1*) (3.6-fold; p_adj_=4.16x10^-13^), semaphorin 3 (*Sema3d*) (10.4-fold; p_adj_=8.03x10^-7^), and neurotransmitter receptor for postsynaptic specialization (*Rapgef4*) (3-fold; p_adj_=0.0003). The downregulation of transcripts coding for neuronal proteins is of interest, because osteocytes express large number of such transcripts (30) and their downregulation may suggest a role for PPARα in maintenance of osteocyte integrity.

### 3.2 Comparison of bone phenotype in osteocyte-specific PPARα knock-out mice vs. global PPARα knock-out mice reveals a regulatory function of PPARα in osteocytes

**Figure 2:**
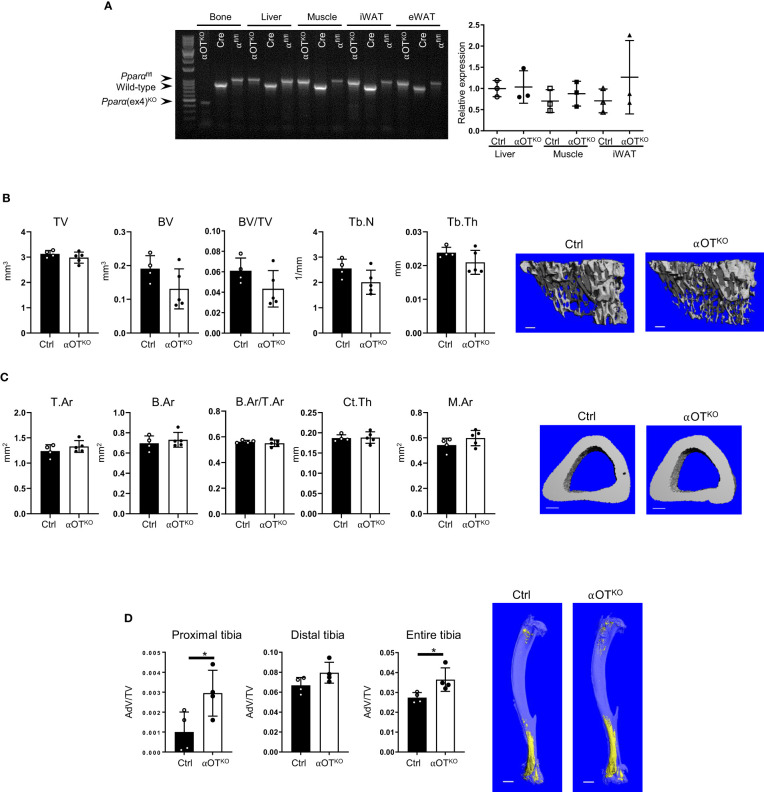
PPARα deletion in osteocytes does not affect trabecular or cortical bone mass but increases bone marrow adipose tissue (BMAT) volume in 6-month-old male mice. **(A)** Analysis of genomic DNA isolated from cortical bone, liver, muscle, inguinal WAT (iWAT), and eWAT of αOT^KO^ and control (Dmp1-Cre and PPARα^flfl^) animals for deletion of exon 4 from the *Pparα* gene locus. *Pparα* DNA fragments of 1070 bp correspond to the floxed allele; those of 915 bp correspond to the wild-type (WT) allele; and those of 450 bp correspond to the allele with deleted exon 4. The graph on the right represents relative mRNA expression of *Pparα* in different tissues of 6-month-old male Ctrl and αOT^KO^ mice. **(B)** Micro-CT renderings and analysis of trabecular bone in proximal tibia: total volume (TV), bone volume (BV), bone mass (BV/TV), trabecular number (Tb.N), and trabecular thickness (Tb.Th). The bar shown on the bone micro-CT renderings corresponds to 0.1 mm. **(C)** Micro-CT renderings and analysis of cortical bone at the tibia midshaft: total cross-sectional area (T.Ar), bone area (B.Ar), bone mass (T.Ar/B.Ar), cortical thickness (Ct.Th), and marrow area (M.Ar). The bar shown on the bone micro-CT renderings corresponds to 0.5 mm. n=4 (Ctrl) and n=5 (αOT^KO^) mice per group. **(D)** Micro-CT renderings and analysis of BMAT volume (AdV/TV) in proximal, distal, and entire tibia stained with osmium tetroxide. The bar shown on the bone micro-CT renderings corresponds to 1 mm. n=4 mice per group. Statistical significance was tested using parametric unpaired Student’s t tests. *p < 0.05.

Mice with osteocyte-specific deletion of PPARα (αOT^KO^) were obtained as a result of breeding 10kb Dmp1-Cre and PPARα^flfl^ mice, as described in the Materials and Methods section. To determine specificity of deletion, sequences encompassing exon 4 were amplified from genomic DNA collected from bone and other tissues using previously described primers (22). Product sizes of 1070 bp, 915 bp, and 450 bp represented a floxed allele, WT allele, and exon 4 deleted allele, respectively. As shown in **Figure 2A**, bone tissue of αOT^KO^ mice harbored deletion of exon 4 from the *Pparα* gene locus. The exon 4 deletion was not detected in liver tissue, muscle, or eWAT, but a faint 450 kb band was observed in iWAT, suggesting that a small fraction of this tissue may also be affected. It has been shown previously that Dmp1-driven Cre recombination may also occur in tissues other than bone, including tissues in which metabolism is under the control of PPARα (31). Therefore, we compared the levels of expression of *Pparα* transcript in liver tissue, muscle, and iWAT of αOT^KO^ mice to the levels of expression in Ctrl mice (**Figure 2A**, right panel). We did not observe significant differences in *Pparα* expression in analyzed tissues isolated from 6-month-old male mice. We have previously shown (in a model of osteocyte-specific PPARγ deletion under the same Dmp1-Cre driver) that, in addition to the abovementioned tissues, *Pparγ* expression is not affected in the cerebellum, small intestine, or kidneys of adult male mice [**Supplementary Table 2** in (23)], suggesting that *Pparα* expression may also not be significantly affected in these tissues of mature αOT^KO^ mice.

Micro-CT analysis of trabecular and cortical bone of 6-month-old αOT^KO^ mice showed no effect of osteocyte-specific deletion of PPARα on bone mass or microarchitecture. Trabecular bone in the proximal tibia was not affected in αOT^KO^ males (**Figure 2B**) or females (**Supplementary Figure 1A**), as compared to controls (Ctrl). Similarly, cortical bone in the midshaft tibia was not affected (**Figure 2C**, **Supplementary Figure 1B**). However, osmium tetroxide (OsO_4_) staining for lipids indicated increased bone marrow adipose tissue (BMAT) volume in the αOT^KO^ tibia, specifically in the proximal part of the male tibia (**Figure 2D**, **Supplementary Figure 1C**). Dynamic and static histomorphometry of trabecular bone in the male tibia did not show any difference in osteoblastic or osteoclastic parameters between αOT^KO^ and Ctrl mice.

**Figure 3:**
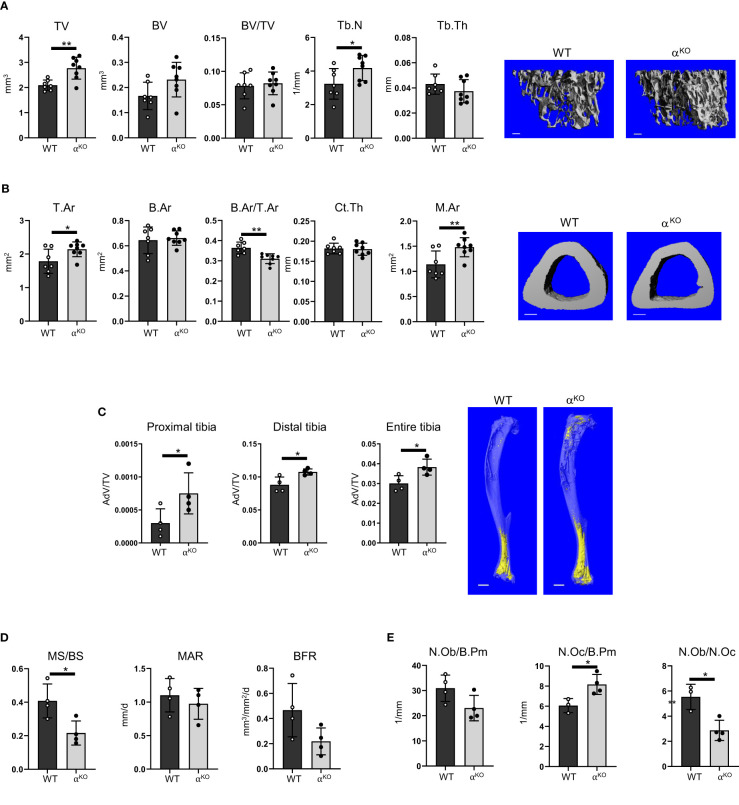
Global deficiency in PPARα affects trabecular and cortical bone, increases BMAT volume, and increases number of osteoclasts in 5-month-old male mice. **(A)** Micro-CT renderings and analysis of trabecular bone in proximal tibia: total volume (TV), bone volume (BV), bone mass (BV/TV), trabecular number (Tb.N), and trabecular thickness (Tb.Th). The bar shown on the bone micro-CT renderings corresponds to 0.1 mm. **(B)** Micro-CT renderings and analysis of cortical bone at the tibia midshaft: total cross-sectional area (T.Ar), bone area (B.Ar), bone mass (B.Ar/T.Ar), cortical thickness (Ct.Th), and marrow area (M.Ar). n=7 (WT) and n=8 (α^KO^) mice per group. The bar shown on the bone micro-CT renderings corresponds to 0.5 mm. **(C)** Micro-CT analysis of BMAT volume (AdV/TV) in proximal, distal, and entire tibia stained with osmium tetroxide. The bar shown on the bone micro-CT renderings corresponds to 1 mm. n=4 mice per group. **(D)** Dynamic histomorphometry of calcein-labeled trabecular bone in proximal tibia, conducted to measure the fraction of mineralizing surface per bone surface (MS/BS), mineral apposition rate (MAR), and bone formation rate (BFR). **(E)** Static histomorphometry of the same tibia bones, evaluating number of osteoblasts and osteoclasts relative to bone perimeter (N.Ob/B.Pm and N.Oc/B.Pm) and osteoblast-to-osteoclast ratio (N.Ob/N.Oc). n=4 mice per group. Statistical significance was tested using parametric unpaired Student’s t tests. *p < 0.05, **p < 0.01.

In contrast, the bone structure of mice with global PPARα deficiency (α^KO^) was affected (**Figure 3**). Trabecular bone in the proximal tibia of 5-month-old α^KO^ males showed an increase in total volume (TV), with a concomitant increase in number of trabeculae (TbN), and a tendency toward increase in trabecular bone volume (BV), but no difference in trabecular bone mass (BV/TV) (**Figure 3A**). In contrast, analysis of cortical bone in the tibia midshaft showed that PPARα deficiency led to bone enlargement (T.Ar), with increased marrow area (M.Ar) but no change in cortical thickness, resulting in a relative decrease in cortical bone mass (B.Ar/T.Ar) (**Figure 3B**). A similar bone phenotype was observed in female mice (**Supplementary Figure 2**). BMAT volume in the tibia of α^KO^ male mice was increased in both proximal and distal locations (**Figure 3C**).

Dynamic histomorphometry showed an overall decrease in mineralized surface (MS/BS) but no changes in mineral apposition rate (MAR) or bone formation rate (BFR) (**Figure 3D**). Osteoblast number was unchanged, but α^KO^ mice had a higher number of osteoclasts, which resulted in an osteoblast/osteoclast ratio favoring bone resorption (**Figure 3E**).

### 3.3 PPARα regulates differentiation of skeletal and hematopoietic stem cells and osteocyte support for BMAT development

**Figure 4:**
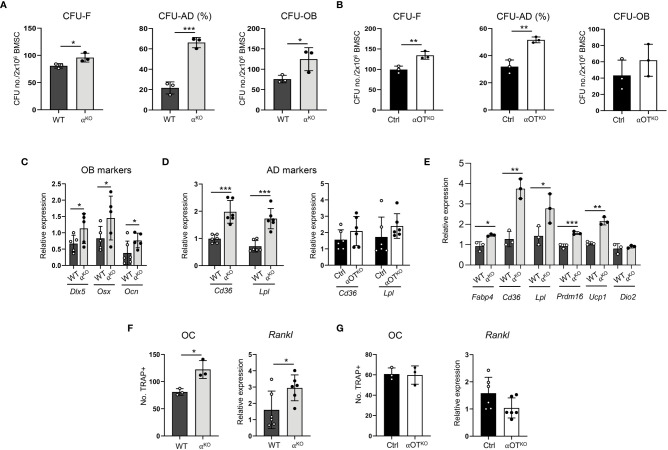
Global and osteocyte-specific deletion of PPARα have different effects on marrow mesenchymal and hematopoietic cell differentiation. **(A, B)** Colony-forming and differentiation potential toward adipocytes and osteoblasts of BMSCs isolated from **(A)** α^KO^ and WT or **(B)** Ctrl and αOT^KO^ mice. Total number of colonies (CFU-F) and number of adipocytic colonies (CFU-AD) were quantified after staining with methyl green and Oil Red O for lipids, and number of osteoblastic colonies (CFU-OB) was quantified after staining with Von Kossa for calcium. **(C)** Relative expressions of osteoblastic gene makers in non-differentiated BMSCs derived from WT and α^KO^ mice. n=5–6 donor mice per group. **(D)** Relative expressions of adipocytic gene markers in non-differentiated BMSCs derived from WT and α^KO^, or Ctrl and αOT^KO^ mice. n=5–6 donor mice per group. **(E)** Expression of adipocyte gene markers in WT BMSCs grown in the presence of conditioned media (CM) collected from primary cultures of either α^KO^ or WT osteocytes (n=3 mice/group). **(F, G)** Differentiation potential of monocytes to osteoclasts (n=3 donor mice per group) and *Rankl* expression in non-differentiated BMSCs. Non-adherent hematopoietic cells from **(F)** WT and α^KO^ mice or **(G)** Ctrl and αOT^KO^ mice were grown in the presence of RANKL (50 ng/ml) and M-CSF (50 ng/ml) for 9 days and stained for tartrate-resistant acid phosphatase (TRAP). TRAP-positive cells with more than 3 nuclei were quantitated as osteoclasts. Statistical significance was tested using parametric unpaired Student’s t tests. *p<0.05, **p<0.01, ***p<0.001.

Growth and differentiation of skeletal stem cells (SSCs) toward osteoblasts and adipocytes was tested in colony-forming unit assays measuring the number of fibroblast (CFU-F), osteoblast (CFU-OB), and adipocyte (CFU-AD) colonies formed *ex vivo* in bone marrow stromal cell (BMSC) cultures derived from α^KO^ and αOT^KO^ animals. As shown in **Figures 4A, B**, both global and osteocyte-specific deficiency in PPARα led to increased potential of SSCs to form CFU-Fs and increased differentiation to adipocytes, as indexed by a higher number of CFU-AD colonies. The cultures of α^KO^ BMSCs (in contrast with BMSC cultures from αOT^KO^ mice) also showed higher number of CFU-OB colonies, which was correlated with increased expression of osteoblast markers, such as *Dlx5*, *Osx*, and *Ocn* in undifferentiated conditions (**Figure 4C**), suggesting a role for PPARα in osteoblast differentiation.

Notably, BMSCs derived from α^KO^ mice exhibited 4-fold higher CFU-AD formation as compared to WT BMSCs, while CFU-AD formation in αOT^KO^ BMSCs was increased only by 40%. In addition, expression of adipocyte-specific gene markers was significantly increased in undifferentiated α^KO^ BMSCs, whereas expression of these markers did not differ from controls in the case of undifferentiated αOT^KO^ BMSCs (**Figure 4D**). These findings suggest that a certain pool of SSCs in the marrow of αOT^KO^ mice is already predetermined for adipocyte differentiation, probably by PPARα-deficient osteocytes. To test for this possibility, we analyzed the effect of conditioned media (CM) collected from primary cultures of PPARα-deficient osteocytes on differentiation of WT BMSCs. As shown in **Figure 4E**, CM derived from PPARα-null osteocytes induced expression of adipocyte-specific markers in BMSCs derived from WT animals, including beige adipocyte markers (*Ucp1* and *Prdm16*). This response points to PPARα-dependent osteocyte paracrine activities regulating SSC allocation to adipocytes, and correlates with the higher volume of BMAT in the proximal tibia of αOT^KO^ male mice.

Analyses of bone structure and histomorphometry suggested increased bone resorption in α^KO^ mice (**Figure 3**). Indeed, osteoclast differentiation from PPARα-null non-adherent bone marrow cells was increased, as well as expression of *Rankl* in BMSCs derived from α^KO^ mice (**Figure 4F**). These effects were not observed in marrow cultures of αOT^KO^ mice. Osteoclast differentiation from non-adherent marrow cells and *Rankl* expression in BMSCs did not differ from Ctrl mice (**Figure 4G**). These findings suggest the conclusion that PPARα negatively controls osteoclast differentiation both directly (at the level of marrow monocyte commitment to osteoclasts) and indirectly (*via* mesenchymal cell support for osteoclastogenesis). Increased hematopoietic cell commitment, together with increased *Rankl* expression in α^KO^ BMSCs, is consistent with the increased number of osteoclasts and enlarged marrow cavities in bone tissue of α^KO^ mice.

### 3.4 Younger mice with either global or osteocyte-specific PPARα deletion exhibit increased energy expenditure

**Figure 5:**
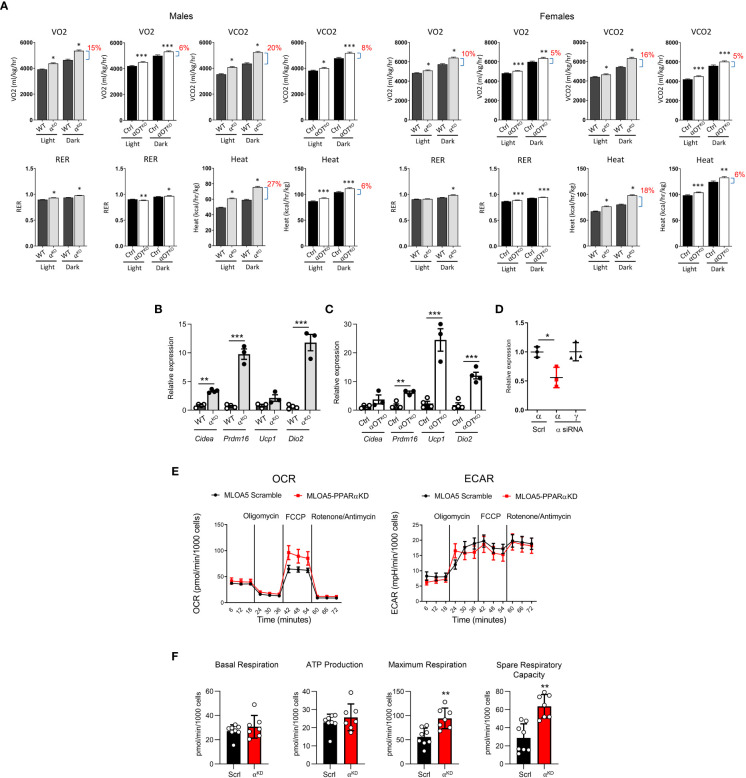
Both global and osteocyte-specific deletion of PPARα lead to increased respiration, increased heat production, and beiging of inguinal white adipose tissue (iWAT) in 5- to 6-month-old male and female mice. **(A)** Metabolic parameters of WT and α^KO^ or Ctrl and αOT^KO^ mice were analyzed for VO2 (oxygen consumption), VCO2 (carbon dioxide production), RER (respiratory exchange ratio), and heat production using the CLAMS system of metabolic cages (n=4 mice/group). Brackets with percentage values (%) represent the fractional increase in the measured parameter. **(B, C)** Relative expression of beige fat markers in iWAT collected from **(B)** WT and α^KO^ male mice or **(C)** Ctrl and αOT^KO^ male mice (n=4 mice/group). **(D)** siRNA silencing of PPARα expression in MLO-A5 osteocytes. α = levels of *Pparα* mRNA; γ = levels of *Pparγ* mRNA; Scrl = scramble siRNA; α siRNA = siRNA specific to PPARα. **(E)** OCR and ECAR profiles in MLO-A5 osteocytes with silenced PPARα. **(F)** Indices of mitochondrial function measured *via* Seahorse Mitochondrial Stress Test. Statistical significance was tested using parametric unpaired Student’s t test. *p<0.05, **p<0.01, ***p<0.001.

Energy expenditure in 5- to 6-month-old α^KO^ and αOT^KO^ mice was measured by indirect calorimetry using the CLAMS system (**Figure 5A**). In both α^KO^ and αOT^KO^ males and females, oxygen consumption (VO2) and carbon dioxide (CO2) production were increased during the daytime and at night, compared to their respective controls. These shifts resulted in an increase in respiratory exchange ratio (RER), indicating increased use of glucose as fuel, and were accompanied by an increase in heat production (**Figure 5A**) and elevated locomotion in males (**Supplementary Figure 3A**), but not in females (**Supplementary Figure 3B**). The body weight of α^KO^ and αOT^KO^ mice did not differ from that of their controls, whereas food intake showed a tendency to increase in αOT^KO^ males and was higher in α^KO^ males (**Supplementary Figures 4A, B**). This was accompanied by beiging of inguinal white adipose tissue (iWAT) in both α^KO^ and αOT^KO^ male mice (**Figures 5B, C**). The increases in energy expenditure parameters observed in αOT^KO^ mice constituted up to 40% of the increases observed in α^KO^ mice, suggesting a significant contribution of osteocytes to global energy metabolism. Thus, deficiency in PPARα, either global or osteocyte-specific, leads to an overall state of elevated thermogenesis with a shift to carbohydrate utilization.

To investigate the function of PPARα in osteocyte bioenergetics, we employed MLO-A5 cells in which PPARα was knocked-down by approximately 50% using siRNA (αA5^KD^ cells) (**Figure 5D**) and analyzed these for mitochondrial activity using the Agilent Seahorse XF Cell Mito Stress test. As shown in **Figure 5E**, oxidative phosphorylation, measured in the form of basal and stressed oxygen consumption rates (OCR), was significantly increased in αA5^KD^ cells. We did not observe differences in extracellular acidification rate (ECAR) between αA5^KD^ and a scrambled control, indicating no effect on anaerobic glycolysis or lactate production (**Figure 5E**). PPARα deficiency did not change basal respiration and ATP production, but did increase maximal cellular respiration in response to stress (**Figure 5F**). This was associated with a significant increase in spare respiratory capacity, indicating that mitochondria might be dysfunctional in PPARα-null osteocytes subjected to certain conditions.

### 3.5 Metabolic phenotype is reverted with aging in both αOT^KO^ and α^KO^ mice, which results in decreased energy expenditure and development of obesity

**Figure 6:**
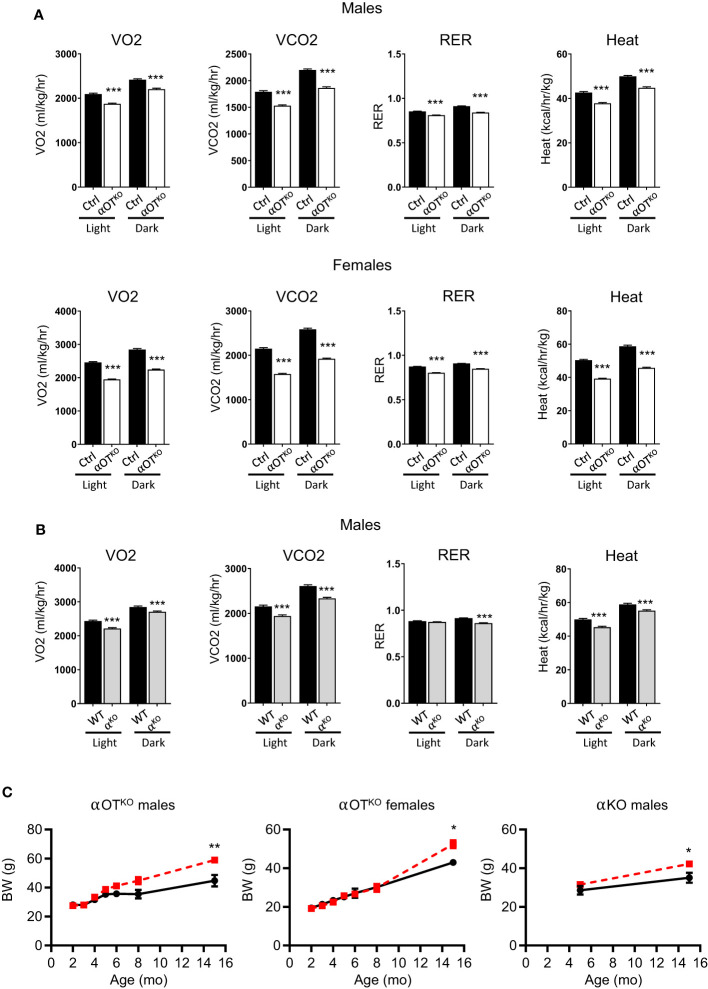
Osteocyte-specific deletion of PPARα reduces metabolic activity and leads to development of obesity in aging mice. **(A)** Metabolic parameters of 15-month-old Ctrl and αOT^KO^ male and female mice, analyzed with the CLAMS system for VO2 (oxygen consumption), VCO2 (carbon dioxide production), RER (respiratory exchange ratio), and heat production (n=4 mice/group). **(B)** Metabolic parameters of 15-month-old WT and α^KO^ male mice analyzed with the CLAMS system, as above. **(C)** Longitudinal comparison of body weight in αOT^KO^ males and females, and α^KO^ males (red dashed line), with control mice (black solid line). Statistical significance was tested using parametric unpaired Student’s t tests. *p<0.05, **p<0.01, ***p<0.001.

The long-term effects of PPARα global and osteocyte-specific deficiency were analyzed in 15-month-old mice. The increased energy expenditure observed in younger αOT^KO^ mice was reversed in aged mice. Both male and female older αOT^KO^ mice showed decreased VO2, VCO2, and RER, and produced less heat (**Figure 6A**). A similar reversal and decrease in energy expenditure was also observed in α^KO^ 15-month-old males (**Figure 6B**). This was accompanied by a significant increase in body weight in both αOT^KO^ and α^KO^ mice (**Figure 6C**), despite either lower (αOT^KO^) or unchanged (α^KO^) food consumption (**Supplementary Figure 4C**). These data suggest that long-term impairment of lipid metabolism due to the absence of PPARα has a negative effect on systemic energy metabolism and obesity development, and that PPARα-deficient osteocytes significantly contribute to this effect. This finding highlights the importance of fatty acid metabolism in osteocytes and the role of osteocytes in maintenance of global energy metabolism.

## 4 Discussion

Although the role of PPARα in fatty acid metabolism is well documented in tissues such as the liver, heart, and muscle, there is a paucity of information on its role in skeletal tissue, which depends heavily on lipid metabolism [reviewed in (13)]. Here, we have demonstrated that PPARα is expressed in osteocytes at levels comparable to those observed in muscle, and regulates the skeletal function of osteocytes and their contribution to systemic energy balance. By performing a side-by-side comparative study of mice with global PPARα deficiency and mice with osteocyte-specific PPARα deficiency, we were able to parse out the different and often subtle activities of PPARα in the skeleton with local and systemic significance.

This study has uncovered the previously unknown contribution of osteocytes to the balance of systemic energy metabolism, in which PPARα plays an important role. At a younger age, both αOT^KO^ and α^KO^ mice demonstrated increased respiration and energy expenditure. The increase in energy production of αOT^KO^ mice constituted up to 40% of that observed in α^KO^ mice. This suggests that, in the state of ambulatory activity, osteocytes contribute significantly to overall energy balance. Increased metabolism was associated with an increased thermogenic phenotype in some fat depots, implying that in addition to lipid metabolism, PPARα also controls osteocyte endocrine activities regulating systemic metabolism. At the level of cellular bioenergetics, PPARα deficiency also increases osteocyte mitochondrial activity under stress, a condition that increases oxidative stress and ROS production, which may have detrimental longitudinal effects. Indeed, and similarly to α^KO^ mice, the αOT^KO^ mice developed obesity with aging and demonstrated a complete reversal in energy expenditure, from a highly energetic state to significantly attenuated expenditure. Thus, initially an absence of PPARα in osteocytes has an additive effect on increased systemic energy metabolism *via* osteocyte bioenergetics and endocrine activities modulating metabolism of peripheral fat, whereas the reversal of this phenotype with aging may reflect prolonged impairment of lipid metabolism and the accumulation of oxidative stress products in bone due to increased mitochondrial activity.

We have shown that, in osteocytes, PPARα regulates a large number of transcripts, many of them coding for secreted proteins involved in the maintenance of bone homeostasis, including signaling proteins of TGFβ/BMP, WNT, FGF, EGF pathways, as well as proteins with a potential role in modulating the bone marrow environment and supporting differentiation niches. However, the bone mass of αOT^KO^ mice was not affected, indicating that either osteocyte activities supporting bone remodeling under PPARα control are of less significance or these activities are counterweighted by other mechanisms. This is in contrast to α^KO^ mice, in which bone phenotype is driven by the absence of PPARα in marrow mesenchymal and hematopoietic cells, which results in changes in bone structure, differentiation potential of bone cells, and increased marrow adiposity. However, regarding marrow adiposity, we have demonstrated that PPARα-controlled osteocyte secretome contributes to the regulation of BMAT development and induction of transcripts in BMSCs coding for proteins involved in fatty acid uptake and thermogenesis. These activities of a paracrine/endocrine character may lead to increased lipid accumulation in bone marrow and increased beiging of marrow and peripheral adipose tissues in a sex-dependent manner.

The complex mode of PPARα function in regulation of bone mass is probably the reason why previous studies were not able to clearly delineate these activities at the organ and cellular levels. The earliest study, performed by Gimble’s group, showed that PPARα-null mice have enlarged marrow cavities (1). Our study has confirmed that α^KO^ mice have enlarged bone cavities and presented evidence that PPARα negatively regulates bone resorption on the level of osteoclastic precursor cells and acts as an inhibitor of *Rankl* expression in marrow stroma cells. These findings are in good agreement with those of several other studies showing a positive effect of fibrates, which are PPARα agonists, on bone mass (2, 3, 32). Most recently, it has been shown that fibrates increase BMP2 production *in vitro* in cells of osteoblastic lineage *via* PPARα acting as a transcriptional regulator of *Bmp2* expression (33). Our transcriptomic studies have shown that, in osteocytes, PPARα acts as a positive regulator of *Bmp2* expression, confirming and expanding the significance of previous findings.

The presented data suggest that PPARα contributes to the maintenance of SSC homeostasis, which is known to be under control of metabolic programing (34). SSCs deficient in PPARα exhibit increased proliferation and increased propensity to differentiate toward both osteoblastic and adipocytic lineages. By juxtaposing analyses of α^KO^ and αOT^KO^ mice, we were able to demonstrate that adipocytic differentiation and SSC proliferation are partially dependent on PPARα function in osteocytes. In contrast, PPARα activities regulating osteoclast differentiation rely mostly on its intrinsic effect in cells of this lineage. In fact, αOT^KO^ mice do not have a distinct bone phenotype, except in relation to increased marrow adiposity, which might be a direct effect of impaired lipid metabolism in osteocytes resulting in increased lipid storage in bone marrow. This finding is concomitant with the observation of enlarged bone marrow cavities, indicating increased bone resorption, in α^KO^ mice but not in αOT^KO^ mice. In conclusion, the role of PPARα in regulation of bone phenotype is rather subtle and is probably accessory to other regulatory pathways. This is in striking contrast to the role of another nuclear receptor, PPARγ, which regulates bone mass on every level, including SSC allocation to the adipocytic lineage at the expense of the osteoblastic lineage, and positive regulation of osteoclastogenesis and sclerostin production in osteocytes (23, 35, 36), making it a more promising target for therapies aiming to increase bone mass. Nevertheless, with the advent of an improved understanding of lipid metabolism in bone, the bone PPARα activities of local and systemic importance also need to be considered.

## Data availability statement

The data presented in the study are deposited in the Gene Expression Ontology (GEO) repository, accession number GSE225595.

## Ethics statement

The animal study was reviewed and approved by University of Toledo Institutional Animal Care and Utilization Committee.

## Author contributions

AC and BL-C designed the experiments; AC conducted the experiments and analyzed the data, including transcriptomic analysis; SB, PC, and EC contributed to the experimental design, data analysis, data interpretation, and editing of manuscript; MC and PG contributed to transcriptomic data analysis; and AC and BL-C wrote the manuscript. All authors take responsibility for the integrity of the data analysis. All authors contributed to the article and approved the submitted version.

## References

[1] Wu X Peters JM Gonzalez FJ Prasad HS Rohrer MD Gimble JM. Frequency of stromal lineage colony forming units in bone marrow of peroxisome proliferator-activated receptor-alpha-null mice. Bone (2000) 26(1):21–6. doi: 10.1016/S8756-3282(99)00238-0 10617153

[2] Samadfam R Awori M Benardeau A Bauss F Sebokova E Wright M. Combination treatment with pioglitazone and fenofibrate attenuates pioglitazone-mediated acceleration of bone loss in ovariectomized rats. J Endocrinol (2012) 212(2):179–86. doi: 10.1530/JOE-11-0356 22062085

[3] Still K Grabowski P Mackie I Perry M Bishop N. The peroxisome proliferator activator receptor alpha/delta agonists linoleic acid and bezafibrate upregulate osteoblast differentiation and induce periosteal bone formation *in vivo* . Calcif Tissue Int (2008) 83(4):285–92. doi: 10.1007/s00223-008-9175-9 18836674

[4] Stunes AK Westbroek I Gustafsson BI Fossmark R Waarsing JH Eriksen EF. The peroxisome proliferator-activated receptor (PPAR) alpha agonist fenofibrate maintains bone mass, while the PPAR gamma agonist pioglitazone exaggerates bone loss, in ovariectomized rats. BMC Endocr Disord (2011) 11:11. doi: 10.1186/1472-6823-11-11 21615901 PMC3127763

[5] Lefebvre P Chinetti G Fruchart JC Staels B. Sorting out the roles of PPAR alpha in energy metabolism and vascular homeostasis. J Clin Invest (2006) 116(3):571–80. doi: 10.1172/JCI27989 PMC1386122 16511589

[6] Rakhshandehroo M Hooiveld G Muller M Kersten S. Comparative analysis of gene regulation by the transcription factor PPARalpha between mouse and human. PloS One (2009) 4(8):e6796. doi: 10.1371/journal.pone.0006796 19710929 PMC2729378

[7] Badman MK Pissios P Kennedy AR Koukos G Flier JS Maratos-Flier E. Hepatic fibroblast growth factor 21 is regulated by PPARalpha and is a key mediator of hepatic lipid metabolism in ketotic states. Cell Metab (2007) 5(6):426–37. doi: 10.1016/j.cmet.2007.05.002 17550778

[8] Inagaki T Dutchak P Zhao G Ding X Gautron L Parameswara V. Endocrine regulation of the fasting response by PPARalpha-mediated induction of fibroblast growth factor 21. Cell Metab (2007) 5(6):415–25. doi: 10.1016/j.cmet.2007.05.003 17550777

[9] Kersten S Seydoux J Peters JM Gonzalez FJ Desvergne B Wahli W. Peroxisome proliferator-activated receptor alpha mediates the adaptive response to fasting. J Clin Invest (1999) 103(11):1489–98. doi: 10.1172/JCI6223 PMC408372 10359558

[10] Guerre-Millo M Rouault C Poulain P Andre J Poitout V Peters JM. PPAR-alpha-null mice are protected from high-fat diet-induced insulin resistance. Diabetes (2001) 50(12):2809–14. doi: 10.2337/diabetes.50.12.2809 11723064

[11] Randle PJ Garland PB Newsholme EA Hales CN. The glucose fatty acid cycle in obesity and maturity onset diabetes mellitus. Ann N Y Acad Sci (1965) 131(1):324–33. doi: 10.1111/j.1749-6632.1965.tb34800.x 5216972

[12] Liu JM Rosen CJ Ducy P Kousteni S Karsenty G. Regulation of glucose handling by the skeleton: Insights from mouse and human studies. Diabetes (2016) 65(11):3225–32. doi: 10.2337/db16-0053 PMC5860442 27959858

[13] Alekos NS Moorer MC Riddle RC. Dual effects of lipid metabolism on osteoblast function. Front Endocrinol (Lausanne) (2020) 11:578194. doi: 10.3389/fendo.2020.578194 33071983 PMC7538543

[14] Niemeier A Niedzielska D Secer R Schilling A Merkel M Enrich C. Uptake of postprandial lipoproteins into bone *in vivo*: impact on osteoblast function. Bone (2008) 43(2):230–7. doi: 10.1016/j.bone.2008.03.022 18538644

[15] Bartelt A Koehne T Todter K Reimer R Muller B Behler-Janbeck F. Quantification of bone fatty acid metabolism and its regulation by adipocyte lipoprotein lipase. Int J Mol Sci (2017) 18(6):1264. doi: 10.3390/ijms18061264 28608812 PMC5486086

[16] Glatz JF Luiken JJ Bonen A. Membrane fatty acid transporters as regulators of lipid metabolism: implications for metabolic disease. Physiol Rev (2010) 90(1):367–417. doi: 10.1152/physrev.00003.2009 20086080

[17] Kevorkova O Martineau C Martin-Falstrault L Sanchez-Dardon J Brissette L Moreau R. Low-bone-mass phenotype of deficient mice for the cluster of differentiation 36 (CD36). PloS One (2013) 8(10):e77701. doi: 10.1371/journal.pone.0077701 24204923 PMC3808405

[18] Kim SP Li Z Zoch ML Frey JL Bowman CE Kushwaha P. Fatty acid oxidation by the osteoblast is required for normal bone acquisition in a sex- and diet-dependent manner. JCI Insight (2017) 2(16):e92704. doi: 10.1172/jci.insight.92704 28814665 PMC5621897

[19] van Gastel N Stegen S Eelen G Schoors S Carlier A Daniels VW. Lipid availability determines fate of skeletal progenitor cells *via* SOX9. Nature (2020) 579(7797):111–7. doi: 10.1038/s41586-020-2050-1 PMC7060079 32103177

[20] Kushwaha P Alekos NS Kim SP Li Z Wolfgang MJ Riddle RC. Mitochondrial fatty acid beta-oxidation is important for normal osteoclast formation in growing female mice. Front Physiol (2022) 13:997358. doi: 10.3389/fphys.2022.997358 36187756 PMC9515402

[21] Bonewald LF. The amazing osteocyte. J Bone Miner Res (2011) 26(2):229–38. doi: 10.1002/jbmr.320 PMC3179345 21254230

[22] Montagner A Polizzi A Fouche E Ducheix S Lippi Y Lasserre F. Liver PPARalpha is crucial for whole-body fatty acid homeostasis and is protective against NAFLD. Gut (2016) 65(7):1202–14. doi: 10.1136/gutjnl-2015-310798 PMC4941147 26838599

[23] Baroi S Czernik PJ Chougule A Griffin PR Lecka-Czernik B. PPARG in osteocytes controls sclerostin expression, bone mass, marrow adiposity and mediates TZD-induced bone loss. Bone (2021) 147:115913. doi: 10.1016/j.bone.2021.115913 33722775 PMC8076091

[24] Bouxsein ML Boyd SK Christiansen BA Guldberg RE Jepsen KJ Muller R. Guidelines for assessment of bone microstructure in rodents using micro-computed tomography. J Bone Miner Res (2010) 25(7):1468–86. doi: 10.1002/jbmr.141 20533309

[25] Liu L Aronson J Huang S Lu Y Czernik P Rahman S. Rosiglitazone inhibits bone regeneration and causes significant accumulation of fat at sites of new bone formation. Calcif Tissue Int (2012) 91(2):139–48. doi: 10.1007/s00223-012-9623-4 PMC3630993 22752619

[26] Dempster DW Compston JE Drezner MK Glorieux FH Kanis JA Malluche H. Standardized nomenclature, symbols, and units for bone histomorphometry: a 2012 update of the report of the ASBMR histomorphometry nomenclature committee. J Bone Miner Res (2013) 28(1):2–17. doi: 10.1002/jbmr.1805 23197339 PMC3672237

[27] Lazarenko OP Rzonca SO Hogue WR Swain FL Suva LJ Lecka-Czernik B. Rosiglitazone induces decreases in bone mass and strength that are reminiscent of aged bone. Endocrinology (2007) 148(6):2669–80. doi: 10.1210/en.2006-1587 PMC2084459 17332064

[28] Huang S Kaw M Harris MT Ebraheim N McInerney MF Najjar SM. Decreased osteoclastogenesis and high bone mass in mice with impaired insulin clearance due to liver-specific inactivation to CEACAM1. Bone (2010) 46(4):1138–45. doi: 10.1016/j.bone.2009.12.020 PMC2862391 20044046

[29] Kramer I Halleux C Keller H Pegurri M Gooi JH Weber PB. Osteocyte wnt/beta-catenin signaling is required for normal bone homeostasis. Mol Cell Biol (2010) 30(12):3071–85. doi: 10.1128/MCB.01428-09 PMC2876685 20404086

[30] Paic F Igwe JC Nori R Kronenberg MS Franceschetti T Harrington P. Identification of differentially expressed genes between osteoblasts and osteocytes. Bone (2009) 45(4):682–92. doi: 10.1016/j.bone.2009.06.010 PMC2731004 19539797

[31] Lim J Burclaff J He G Mills JC Long F. Unintended targeting of Dmp1-cre reveals a critical role for Bmpr1a signaling in the gastrointestinal mesenchyme of adult mice. Bone Res (2017) 5:16049. doi: 10.1038/boneres.2016.49 28163952 PMC5282469

[32] Smith SY Samadfam R Chouinard L Awori M Benardeau A Bauss F. Effects of pioglitazone and fenofibrate co-administration on bone biomechanics and histomorphometry in ovariectomized rats. J Bone Miner Metab (2015) 33(6):625–41. doi: 10.1007/s00774-014-0632-4 25534548

[33] Kim YH Jang WG Oh SH Kim JW Lee MN Song JH. Fenofibrate induces PPARalpha and BMP2 expression to stimulate osteoblast differentiation. Biochem Biophys Res Commun (2019) 520(2):459–65. doi: 10.1016/j.bbrc.2019.10.048 31607484

[34] Tencerova M Rendina-Ruedy E Neess D Faergeman N Figeac F Ali D. Metabolic programming determines the lineage-differentiation fate of murine bone marrow stromal progenitor cells. Bone Res (2019) 7:35. doi: 10.1038/s41413-019-0076-5 31754546 PMC6856123

[35] Shockley KR Lazarenko OP Czernik PJ Rosen CJ Churchill GA Lecka-Czernik B. PPARg2 nuclear receptor controls multiple regulatory pathways of osteoblast differentiation from marrow mesenchymal stem cells. J Cell Biochem (2009) 106(2):232–46. doi: 10.1002/jcb.21994 PMC2745312 19115254

[36] Stechschulte LA Czernik PJ Rotter ZC Tausif FN Corzo CA Marciano DP. PPARG post-translational modifications regulate bone formation and bone resorption. EBioMedicine (2016) 10:174–84. doi: 10.1016/j.ebiom.2016.06.040 PMC5006645 27422345

