# Supplementary material for: Osteocytes contribute via nuclear receptor PPAR-alpha to maintenance of bone and systemic energy metabolism
Source: Front Endocrinol (Lausanne). 2023 Apr 18;14:1145467. doi: 10.3389/fendo.2023.1145467 (PMC10173151; doi:10.3389/fendo.2023.1145467)
Supplement: Supplementary file 1 [file Presentation_1.pptx]

## Slide 1
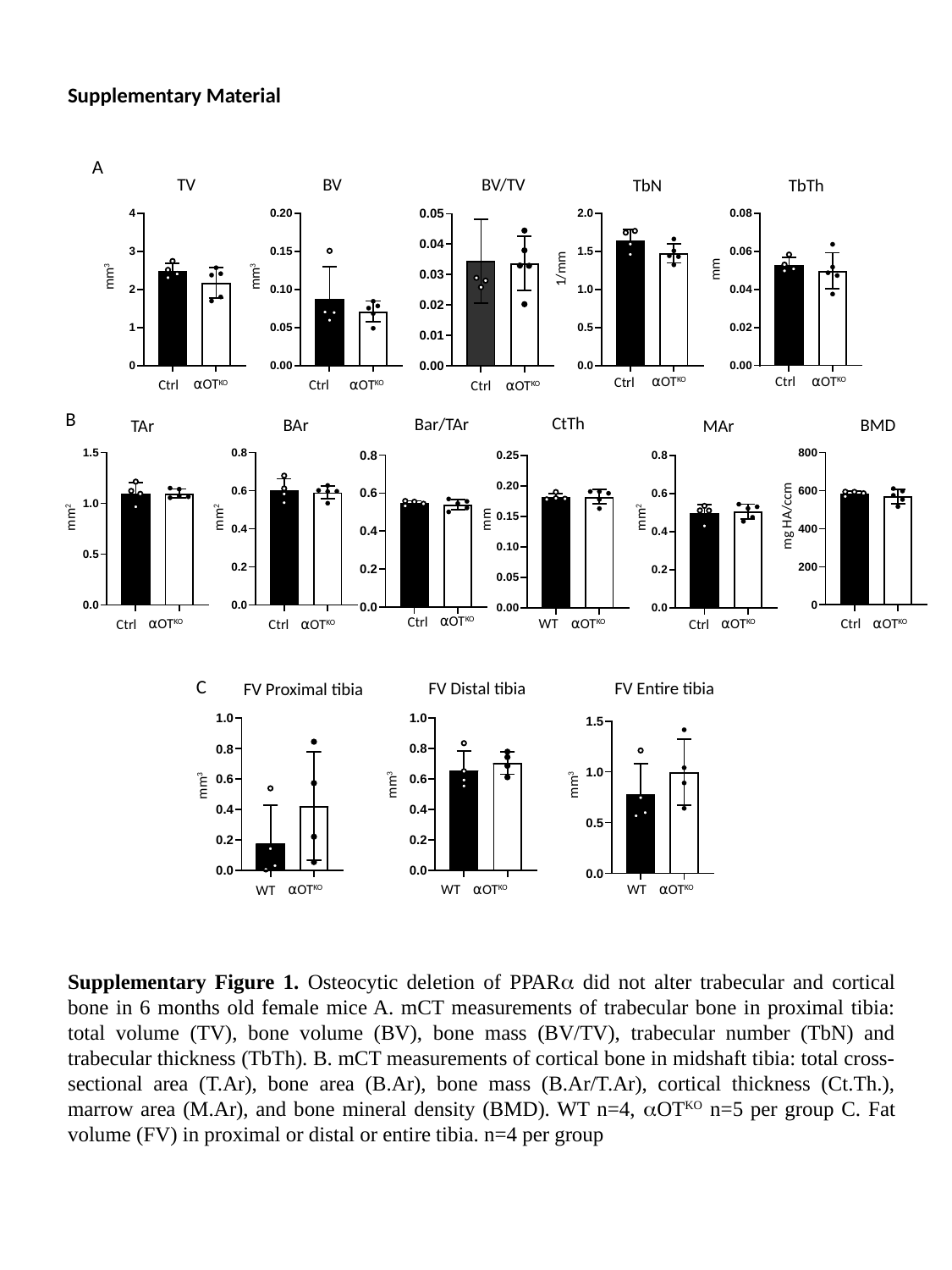

Supplementary Material
A
BV
mm3
⍺OTKO
Ctrl
TV
mm3
⍺OTKO
Ctrl
BV/TV
⍺OTKO
Ctrl
TbN
1/mm
⍺OTKO
Ctrl
TbTh
mm
⍺OTKO
Ctrl
B
CtTh
mm
⍺OTKO
WT
Bar/TAr
⍺OTKO
Ctrl
BAr
mm2
⍺OTKO
Ctrl
BMD
mg HA/ccm
⍺OTKO
Ctrl
TAr
mm2
⍺OTKO
Ctrl
MAr
mm2
⍺OTKO
Ctrl
C
FV Entire tibia
FV Distal tibia
FV Proximal tibia
mm3
mm3
mm3
⍺OTKO
⍺OTKO
WT
WT
⍺OTKO
WT
Supplementary Figure 1. Osteocytic deletion of PPARα did not alter trabecular and cortical bone in 6 months old female mice A. mCT measurements of trabecular bone in proximal tibia: total volume (TV), bone volume (BV), bone mass (BV/TV), trabecular number (TbN) and trabecular thickness (TbTh). B. mCT measurements of cortical bone in midshaft tibia: total cross-sectional area (T.Ar), bone area (B.Ar), bone mass (B.Ar/T.Ar), cortical thickness (Ct.Th.), marrow area (M.Ar), and bone mineral density (BMD). WT n=4, αOTKO n=5 per group C. Fat volume (FV) in proximal or distal or entire tibia. n=4 per group

## Slide 2
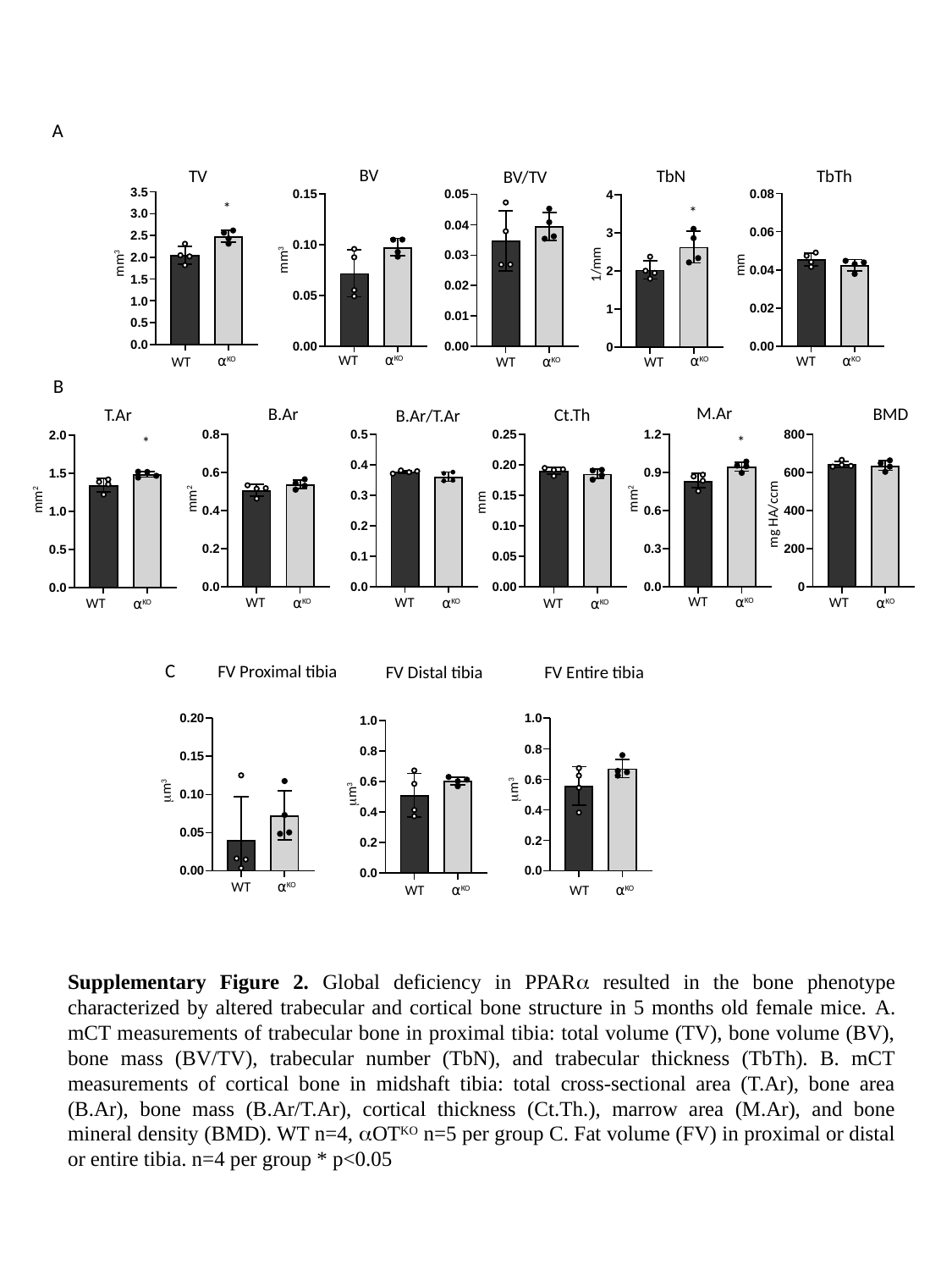

A
BV
mm3
⍺KO
WT
TbTh
mm
⍺KO
WT
TV
*
mm3
⍺KO
WT
TbN
*
1/mm
⍺KO
WT
BV/TV
⍺KO
WT
B
M.Ar
*
mm2
WT
⍺KO
B.Ar
mm2
WT
⍺KO
T.Ar
*
mm2
WT
⍺KO
Ct.Th
mm
WT
⍺KO
B.Ar/T.Ar
WT
⍺KO
C
FV Proximal tibia
FV Distal tibia
FV Entire tibia
mm3
mm3
mm3
⍺KO
WT
WT
⍺KO
⍺KO
WT
Supplementary Figure 2. Global deficiency in PPARα resulted in the bone phenotype characterized by altered trabecular and cortical bone structure in 5 months old female mice. A. mCT measurements of trabecular bone in proximal tibia: total volume (TV), bone volume (BV), bone mass (BV/TV), trabecular number (TbN), and trabecular thickness (TbTh). B. mCT measurements of cortical bone in midshaft tibia: total cross-sectional area (T.Ar), bone area (B.Ar), bone mass (B.Ar/T.Ar), cortical thickness (Ct.Th.), marrow area (M.Ar), and bone mineral density (BMD). WT n=4, αOTKO n=5 per group C. Fat volume (FV) in proximal or distal or entire tibia. n=4 per group * p<0.05
BMD
mg HA/ccm
WT
⍺KO

## Slide 3
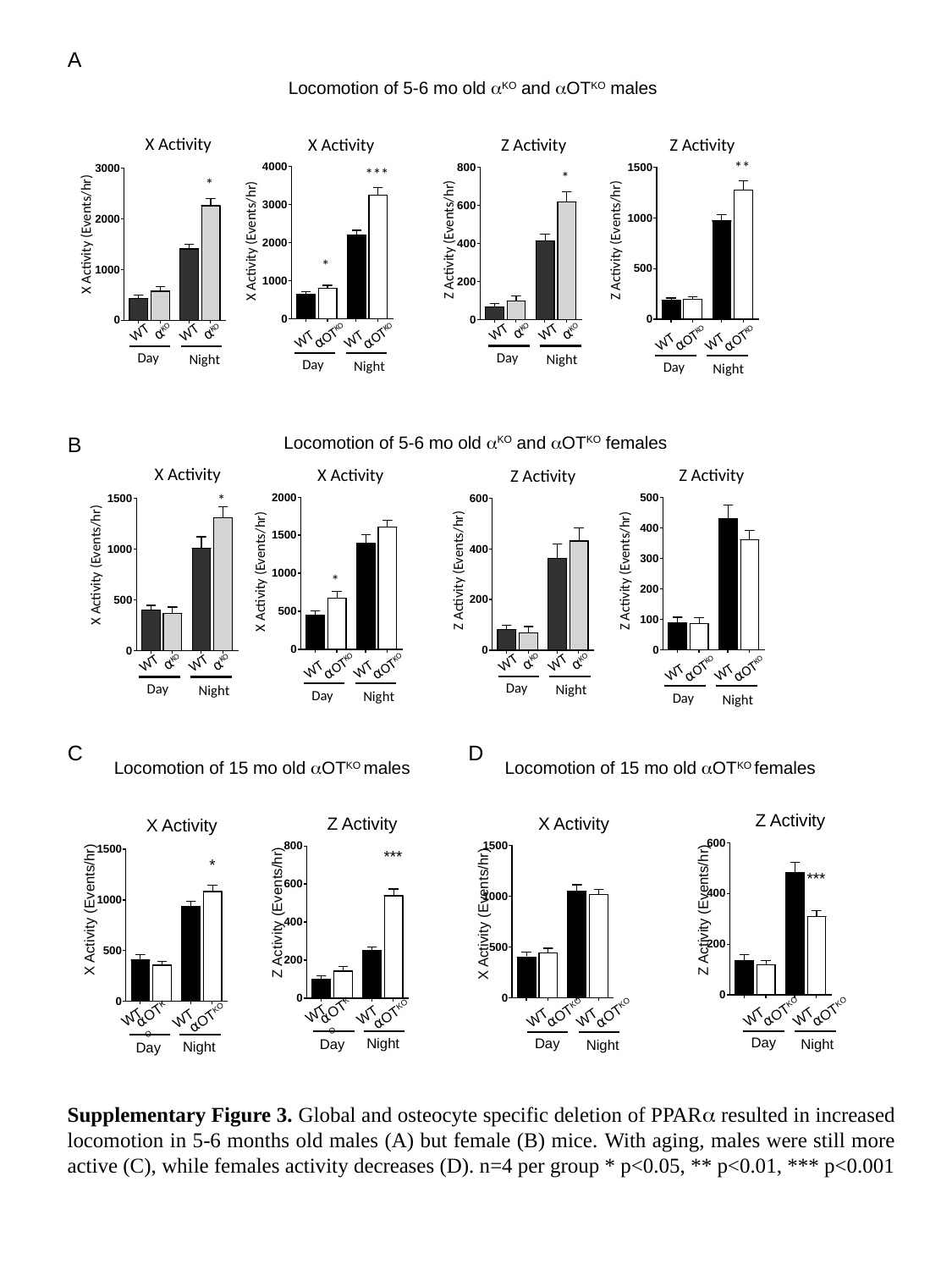

A
Locomotion of 5-6 mo old αKO and αOTKO males
X Activity
X Activity (Events/hr)
⍺KO
⍺KO
WT
WT
Day
Night
Z Activity
Z Activity (Events/hr)
⍺OTKO
⍺OTKO
WT
WT
Day
Night
X Activity
X Activity (Events/hr)
⍺OTKO
⍺OTKO
WT
WT
Day
Night
Z Activity
Z Activity (Events/hr)
⍺KO
⍺KO
WT
WT
Day
Night
**
***
*
*
*
B
Locomotion of 5-6 mo old αKO and αOTKO females
X Activity
*
X Activity (Events/hr)
⍺KO
⍺KO
WT
WT
Day
Night
Z Activity
Z Activity (Events/hr)
⍺OTKO
⍺OTKO
WT
WT
Day
Night
X Activity
X Activity (Events/hr)
*
⍺OTKO
⍺OTKO
WT
WT
Day
Night
Z Activity
Z Activity (Events/hr)
⍺KO
⍺KO
WT
WT
Day
Night
C
D
Locomotion of 15 mo old αOTKO females
Locomotion of 15 mo old αOTKO males
Z Activity
Z Activity (Events/hr)
⍺OTKO
⍺OTKO
WT
WT
Day
Night
***
Z Activity
Z Activity (Events/hr)
***
⍺OTKO
WT
WT
⍺OTKO
Day
Night
X Activity
X Activity (Events/hr)
⍺OTKO
⍺OTKO
WT
WT
Day
Night
X Activity
X Activity (Events/hr)
*
⍺OTKO
WT
WT
⍺OTKO
Day
Night
Supplementary Figure 3. Global and osteocyte specific deletion of PPARα resulted in increased locomotion in 5-6 months old males (A) but female (B) mice. With aging, males were still more active (C), while females activity decreases (D). n=4 per group * p<0.05, ** p<0.01, *** p<0.001

## Slide 4
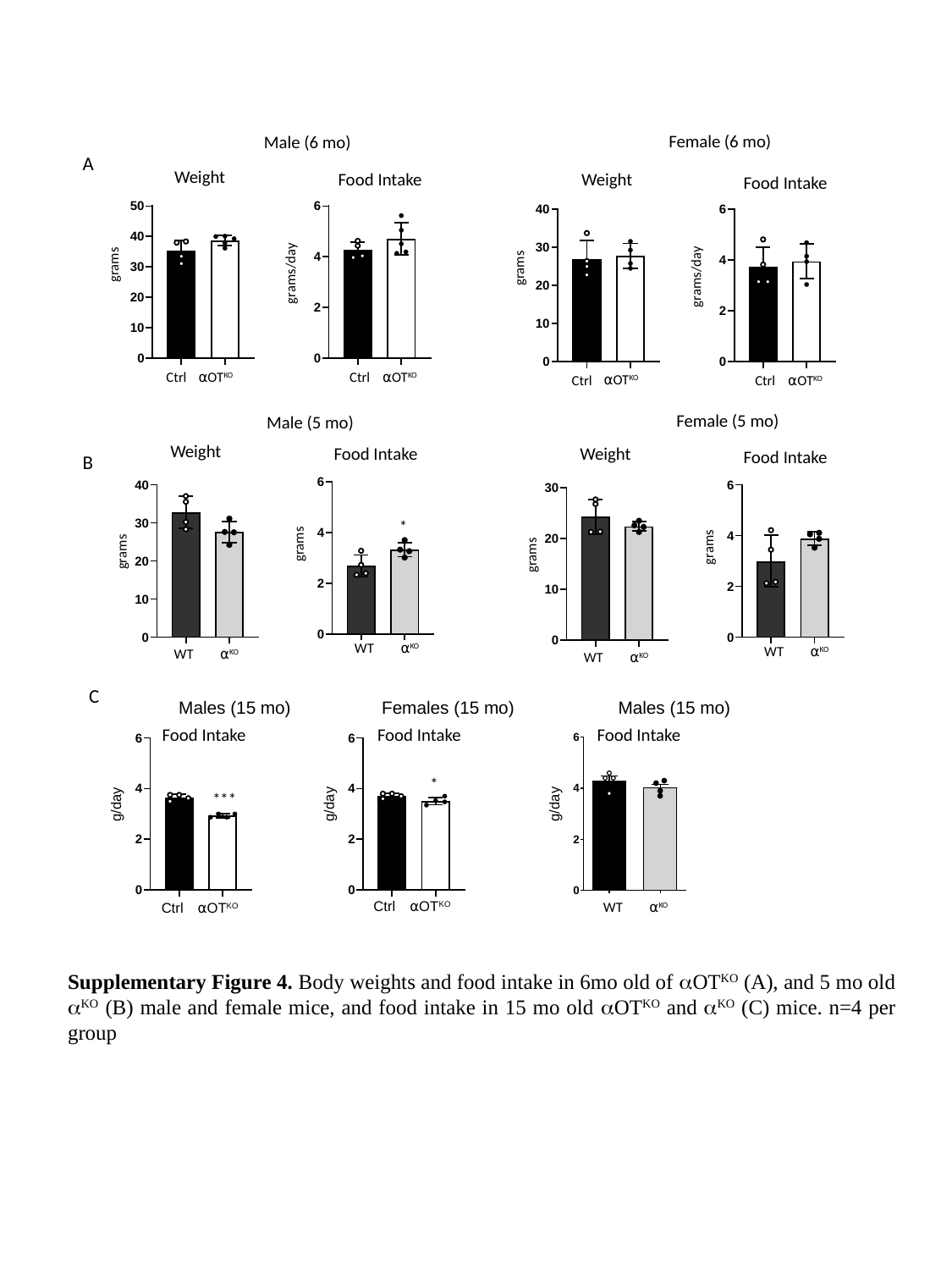

Female (6 mo)
Male (6 mo)
A
Weight
Food Intake
Weight
Food Intake
grams
grams/day
grams
grams/day
⍺OTKO
Ctrl
⍺OTKO
Ctrl
⍺OTKO
Ctrl
⍺OTKO
Ctrl
Female (5 mo)
Male (5 mo)
Weight
Weight
Food Intake
Food Intake
B
*
grams
grams
grams
grams
⍺KO
WT
⍺KO
WT
⍺KO
WT
⍺KO
WT
C
Males (15 mo)
Females (15 mo)
Males (15 mo)
Food Intake
Food Intake
Food Intake
*
***
g/day
g/day
g/day
Ctrl
⍺OTKO
⍺KO
WT
Ctrl
⍺OTKO
Supplementary Figure 4. Body weights and food intake in 6mo old of αOTKO (A), and 5 mo old αKO (B) male and female mice, and food intake in 15 mo old αOTKO and αKO (C) mice. n=4 per group
