# Supplementary material for: Osteocytes contribute via nuclear receptor PPAR-alpha to maintenance of bone and systemic energy metabolism
Source: Front Endocrinol (Lausanne). 2023 Apr 18;14:1145467. doi: 10.3389/fendo.2023.1145467 (PMC10173151; doi:10.3389/fendo.2023.1145467)
Supplement: Supplementary file 2 [file Presentation_2.pptx]

## Slide 1
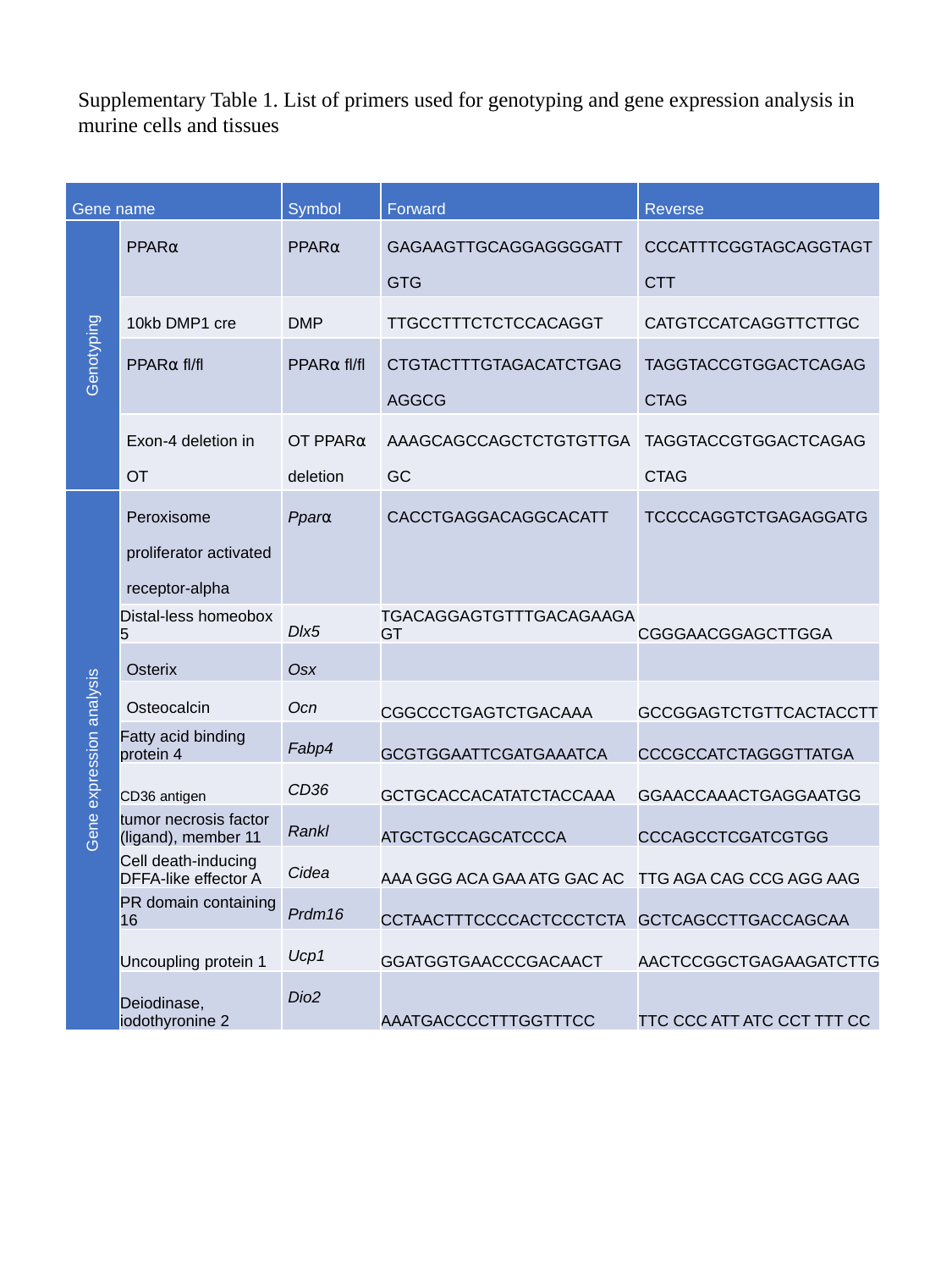

Supplementary Table 1. List of primers used for genotyping and gene expression analysis in murine cells and tissues
| Gene name | | Symbol | Forward | Reverse |
| --- | --- | --- | --- | --- |
| Genotyping | PPAR⍺ | PPAR⍺ | GAGAAGTTGCAGGAGGGGATT GTG | CCCATTTCGGTAGCAGGTAGTCTT |
| | 10kb DMP1 cre | DMP | TTGCCTTTCTCTCCACAGGT | CATGTCCATCAGGTTCTTGC |
| | PPAR⍺ fl/fl | PPAR⍺ fl/fl | CTGTACTTTGTAGACATCTGAGAGGCG | TAGGTACCGTGGACTCAGAGCTAG |
| | Exon-4 deletion in OT | OT PPAR⍺ deletion | AAAGCAGCCAGCTCTGTGTTGAGC | TAGGTACCGTGGACTCAGAGCTAG |
| Gene expression analysis | Peroxisome proliferator activated receptor-alpha | Ppar⍺ | CACCTGAGGACAGGCACATT | TCCCCAGGTCTGAGAGGATG |
| | Distal-less homeobox 5 | Dlx5 | TGACAGGAGTGTTTGACAGAAGAGT | CGGGAACGGAGCTTGGA |
| | Osterix | Osx | | |
| | Osteocalcin | Ocn | CGGCCCTGAGTCTGACAAA | GCCGGAGTCTGTTCACTACCTT |
| | Fatty acid binding protein 4 | Fabp4 | GCGTGGAATTCGATGAAATCA | CCCGCCATCTAGGGTTATGA |
| | CD36 antigen | CD36 | GCTGCACCACATATCTACCAAA | GGAACCAAACTGAGGAATGG |
| | tumor necrosis factor (ligand), member 11 | Rankl | ATGCTGCCAGCATCCCA | CCCAGCCTCGATCGTGG |
| | Cell death-inducing DFFA-like effector A | Cidea | AAA GGG ACA GAA ATG GAC AC | TTG AGA CAG CCG AGG AAG |
| | PR domain containing 16 | Prdm16 | CCTAACTTTCCCCACTCCCTCTA | GCTCAGCCTTGACCAGCAA |
| | Uncoupling protein 1 | Ucp1 | GGATGGTGAACCCGACAACT | AACTCCGGCTGAGAAGATCTTG |
| | Deiodinase, iodothyronine 2 | Dio2 | AAATGACCCCTTTGGTTTCC | TTC CCC ATT ATC CCT TTT CC |
